# A Multiscale Method for Infrared Ship Detection Based on Morphological Reconstruction and Two-Branch Compensation Strategy

**DOI:** 10.3390/s23167309

**Published:** 2023-08-21

**Authors:** Xintao Chen, Changzhen Qiu, Zhiyong Zhang

**Affiliations:** School of Electronics and Communication Engineering, Sun Yat-sen University, Shenzhen 518107, China

**Keywords:** infrared image, ship target detection, morphological reconstruction, contour-based templates, multiscale detection

## Abstract

Infrared ship target detection is crucial technology in marine scenarios. Ship targets vary in scale throughout navigation because the distance between the ship and the infrared camera is constantly changing. Furthermore, complex backgrounds, such as sea clutter, can cause significant interference during detection tasks. In this paper, multiscale morphological reconstruction-based saliency mapping, combined with a two-branch compensation strategy (MMRSM-TBC) algorithm, is proposed for the detection of ship targets of various sizes and against complex backgrounds. First, a multiscale morphological reconstruction method is proposed to enhance the ship targets in the infrared image and suppress any irrelevant background. Then, by introducing a structure tensor with two feature-based filter templates, we utilize the contour information of the ship targets and further improve their intensities in the saliency map. After that, a two-branch compensation strategy is proposed, due to the uneven distribution of image grayscale. Finally, the target is extracted using an adaptive threshold. The experimental results fully show that our proposed algorithm achieves strong performance in the detection of different-sized ship targets and has a higher accuracy than other existing methods.

## 1. Introduction

With the advantages of small size, a simple structure, high concealment, a long detection distance, and all-weather operation, infrared imaging technology has been widely used in various fields. Among these uses, infrared ship detection has significant value [1,2]. Infrared ship detection plays a crucial role in civilian fields, such as in maritime transportation, fishery management, and ocean rescue. The role of infrared ship detection in military fields, such as in naval construction and illegal immigration surveillance, is also irreplaceable. Therefore, in recent years, infrared ship target detection has been a research hotspot.

Although infrared monitoring systems can monitor sea surface conditions twenty-four hours a day every day of the week, without being affected by darkness and harsh environments, infrared imaging generally suffers from characteristics, such as a narrow grayscale range, low contrast, and a low signal-to-noise ratio. At the same time, due to factors, such as weather conditions, complex image scenes, and sea winds and waves, complex sea surface backgrounds inevitably have strong fluctuating clutter and dynamic noise, which increase the difficulty of infrared target detection and tracking. Thus, infrared ship target detection is a challenging task. Furthermore, the scale issue in infrared imaging also poses certain challenges for detection. Therefore, this paper is devoted to solving the problems caused by inconsistent target sizes and complex backgrounds in infrared ship target detection.

In the past few decades, researchers have developed many methods for infrared ship target detection, including methods based on the following: local contrast measure (LCM) [1,3,4,5,6], threshold segmentation [7,8,9], background modeling [10,11], deep learning [12,13,14,15], and morphological reconstruction [16,17].

Methods based on local contrast measure [4] have been widely used for detecting small infrared targets in recent years. Zhang et al. [1] designed a local patch-based similarity description operator to represent the similarity between local image patches by making full use of the differences in spatial structure between wave and target. In [3], a multiscale candidate target map was constructed by using a Gabor filter to extract the edge subband and to calculate the intensity difference between the central block and its neighbour blocks. To enhance the small target, Cui et al. [5] proposed a weighted three-layer window local contrast approach. However, this approach has a drawback in that the size of the window is fixed and cannot be adjusted for targets with different sizes. An adaptive local gradient variation was developed by Ping et al, to improve local contrast for infrared ship targets [6]. The algorithm works well in finding small targets when the background is smooth. However, the accuracy of this approach can easily be reduced if the background noise is complex, such as if, for example, it contains dazzling wave noise. Small targets can be efficiently enhanced by using local contrast-based methods; these methods usually require the detected targets to have a small size and a gray distribution that is uniform and distinguishable from the surrounding environment.

There are also many methods based on threshold segmentation that have proven to be efficient when detecting infrared ship targets. This kind of method has advantages in that it is simple and easy to implement. Classical threshold segmentation methods, such as Otsu [7], have been widely used in the field of detection. However, classical methods are too simple and cannot cope with noise interference. For example, when there is strong sea clutter in an image, Otsu is not able to segment the image correctly. Active contour-based Chan–Vese models [8,9] are also commonly utilized in the field of ship target segmentation. Unfortunately, when the ship targets have low contrast compared to the background, such as in scenarios in which ships are submerged in severe sea clutter, the performance of these methods suffers greatly.

The background modeling technology consists of two stages. First, it obtains a background estimation from the sequences and, then, segments the foreground by subtracting the background from the image. By using the information of multiframe images, this technology can suppress the background more thoroughly, compared to single-frame detection methods. Zhou et al. [10] modeled the fluctuating sea background in the Fourier frequency domain and achieved considerable accuracy in the background model and the separability of the target and the background. In [11], a method based on background modeling, combined with multiple features, was proposed, and two strategies were utilized to update the background for two different cases that might appear in two adjacent frames. Although these background modeling-based methods can take full advantage of the spatial–frequential information in the image sequence, they are multiframe-based and consume a lot of resources. Furthermore, the methods manually set too many parameters, which is not conducive to practical applications.

Deep learning-based detection methods have received widespread attention because of their high accuracy, concurrent with a continuous improvement in computer performance. Zhou et al. [12] presented a straightforward one-stage ship recognition network to learn joint features from multi-resolution infrared pictures for greater accuracy and resilience in large-scale infrared images. Liu et al. [13] employed a two-branch structure, consisting of a contour prediction branch and a saliency map generation branch, to fully leverage edge information. The proposed network has improved accuracy and, at the same time, fewer parameters when detecting infrared ship targets. Wang et al. [14] proposed an inverse optical flow method to estimate the candidate targets’ local images, and a depth normalization, wherein the position of the sea–sky line is built on the basis of the principle of the projection perspective. For the purpose of identifying marine ship targets, Liu et al. introduced a cross-layer multi-task convolutional neural network model [15]. During the training stage, 2420 RGB photos were utilized for training, and for model testing, they used 631 RGB images of the same quality, achieving a satisfactory result. However, this technique requires high picture quality and important information, such as object borders and contours and color information, to extract deep level features. Therefore, infrared images with poor contrast, a hazy target outline, and a low signal-to-noise ratio are not ideal training data for deep-learning-based methods. In fact, the advanced deep learning-based methods for detecting ship targets mainly focus on two domains: detecting ship targets from RGB images with rich color information and high contrast [15], and detecting ship targets from remote sensing images, in which the targets have obvious features and there is less interference, such as sea clutter in the remote sensing images [12,18,19]. However, for infrared images taken by infrared cameras, the advantages mentioned above do not exists. The size of the dataset can be another problem. The training stage of a deep learning-based method requires thousands of images to ensure the model learns sufficient information about the targets, and the corresponding manually annotated images can also be enormous in quantity, which is a great challenge for both manpower and resources.

Morphological reconstruction (MR) is a powerful operation in mathematical morphology [20]. In the decades since its introduction, scholars have applied it to numerous fields related to image processing and have achieved remarkable results. For example, morphological reconstruction has now been widely used in image filtering [21], image segmentation [22], and feature extraction [23]. A significantly fast and robust algorithm for image segmentation was proposed in [24], utilizing the local spatial information. Lei et al. [25] proposed an adaptive morphological reconstruction algorithm, achieving a state-of-the-art performance in seeded image segmentation. Wang et al. [26] utilized morphological reconstruction in their Fuzzy C Means-based algorithm to improve Fuzzy C Means’ robustness and to enable the distribution characteristics of image pixels to be favorable for fuzzy clustering. In [16], Li et al. proposed a method to detect ship targets in a severely cluttered background, based on the theory of morphological reconstruction and a multi-feature analysis. However, a flaw of this method is that the features of the ship are variable and it is hard to determine a common feature for all ships. Furthermore, the performance of this method falls when the targets’ intensities are uneven. In [17], a robust method was proposed to detect infrared targets in low-contrast environments. This method utilizes morphological reconstruction to enhance targets, and, by introducing an entropy-based thresholding approach, the targets can be extracted. This method works well when detecting low contrast infrared targets, but if the background is complicated, such as when, for example, it contains mountains and shores, it may misclassify the background as a true target.

Based on the analysis above, we can summarize the current problems in infrared ship detection tasks. First, infrared cameras are usually fixed in position when shooting, while ships are often in a moving state. The relative distance between the infrared camera and the ship is constantly changing and the size of the ship in the infrared image captured by the camera is not fixed; therefore, existing single-scale methods are not appropriate in this situation. Furthermore, in infrared images, ship targets may have low contrast, due to the influence of weather and lighting, which leads to poor distinguishability between the ships and their surrounding environments, making it more difficult to segment the targets from the background. In this case, methods based on local contrast cannot achieve good performance. In addition, the shape and gray distribution of backgrounds, such as those of mountains and coasts, may be similar to the target ship and they may not be correctly distinguished from the target in a saliency map; thereby affecting the segmentation results. In this case, methods based on specific shape features may possibly fail.

To address the potential issues mentioned above and to achieve better detection results, we propose an infrared ship detection algorithm based on multiscale morphological reconstruction saliency mapping, combined with a two-branch compensation strategy (MMRSP-TBC). The main contributions of our work are as follows:

(1) Taking advantage of morphological reconstruction’s outstanding performance in image processing, we use it to remove noise from images and to smooth complex clutter interference in the background in the image preprocessing stage. We, then, incorporate multiscale methods into this step to make it effective for targets of different scales.

(2) We introduce a feature-based structural tensor into our method. We designed two feature-based templates to convolve them with the structural tensor matrix. This shape feature has universal adaptability, as it utilizes the edge undulation characteristics of ships. By incorporating this method, the intensity of the ship in the image is further enhanced and the contrast with the background is further improved.

(3) A two-branch compensation strategy, that utilizes the significant shape characteristics of ships, is proposed to guide the segmentation process; thereby improving the segmentation results.

The remainder of the paper is formed as follows. Section 2 provides a brief introduction to morphological reconstruction and demonstrates the proposed algorithm. Section 3 analyzes and selects the hyperparameters used in the algorithm and presents the experimental results. Section 4 summarizes the paper.

## 2. Materials and Methods

**Figure 1:**
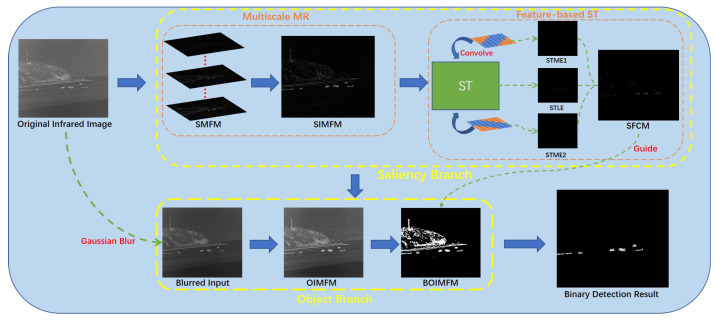
The overall procedure of the proposed method.

The framework of the proposed method is summarized in Figure 1. Based on morphological reconstruction, we propose a multiscale method to overcome the potential issues caused by scale inconsistencies. Then, we apply two templates, based on the common features of the ship, to the structure tensor to make the ship target more prominent in the saliency map. Finally, a two-branch compensation strategy is introduced to solve the problem of incomplete segmentation caused by uneven gray distribution in the infrared image.

To be specific, our proposed method first processes the original infrared image with the multiscale morphological reconstruction (MMR) method. Then, by integrating the foreground map of different scales, we obtain the integrated multiscale foreground map (IMFM). After that, we initialize the feature-based templates and convolve them with the structure tensor (ST) matrix to obtain the feature-based structure tensor. We, first, obtain the saliency branch feature-based contour map (SFCM) as an indicator to guide the segmentation process and its maximum value M is used as a judging threshold. For the object branch, we process the original image with Gaussian blur and obtain a blurred input. We implement the same method on the blurred image as on the saliency branch and derive the object branch integrated multiscale foreground map (OIMFM), and its binarized result, BOIMFM. It is now possible to detect targets from BOIMFM and we process the targets separately to determine whether they are the true target. For each target in BOIMFM, we determine it to be a true target only when its corresponding average value of SFCM is larger than 0.5*M. A target having an average SFCM that does not satisfy this criterion is judged to be a false target. Then, the targets remaining in the binarized image are the final true targets detected by our method.

### 2.1. Morphological Reconstruction

Based on mathematical morphological operations, grayscale morphological reconstruction is used to solve problems, such as filtering and segmentation [27]. In the process of morphological reconstruction, two images are needed: a marker image and a mask image. The marker image is considered to be the original image and is iteratively reconstructed under the constraint of the mask image. The iteration continues until the image value is stable. Given a structuring element *B*, a marker image *f*, and a mask image *g*, an elementary geodesic dilation of size 1 is then defined as follows:

(1) $$D_{g}^{\left(1\right)}\left(f\right)=\left(f⊕B\right)\wedge g$$

where ⊕ is the basic morphological dilation operator and ∧ is the pointwise minimum operator.

Then, a grayscale geodesic dilation of size j $\left(j\geq 2\right)$ is given in Equation (2).

(2) $$D_{g}^{\left(j\right)}\left(f\right)=D_{g}^{\left(1\right)}\left(D_{g}^{\left(j-1\right)}\left(f\right)\right).$$

With the deduction above, we can obtain an expression for dilation-based reconstruction (DR) of *f* from *g* when $D_{g}^{\left(j\right)}\left(f\right)=D_{g}^{\left(j-1\right)}\left(f\right)$, which means stability is reached.

(3) $$DR_{g}\left(f\right)=D_{g}^{\left(j\right)}\left(f\right)$$

Similarly, a size 1 elementary geodesic erosion is given as follows:

(4) $$E_{g}^{\left(1\right)}\left(f\right)=\left(f\Theta B\right)\vee g$$

where Θ is the basic morphological erosion operator and ∨ represents the pointwise maximum operation.

When stability is reached, denoted as $E_{g}^{\left(j\right)}\left(f\right)=E_{g}^{\left(j-1\right)}\left(f\right)$, the erosion-based reconstruction (ER) of mask *g* from marker *f* is given by:

(5) $$ER_{g}\left(f\right)=E_{g}^{\left(j\right)}\left(f\right)$$

Morphological opening and closing operations exhibit a great performance for image restoration and feature extraction [25]. For example, they restore the forms of objects that the structuring element exceeds after each process [27]. In [16], Li et al. utilized the opening and closing results of the original grayscale image as the marker image and achieved a significant result in maritime target detection. In this paper, we follow this method of choosing the result of the opening operation of the grayscale image *g*, denoted as $f_{d}=g⊕B$, as the marker image in dilation-based reconstruction and the result of the closing operation of *g*, denoted as $f_{e}=g⊕B$, as the marker image in the erosion-based reconstruction process.

**Figure 2:**
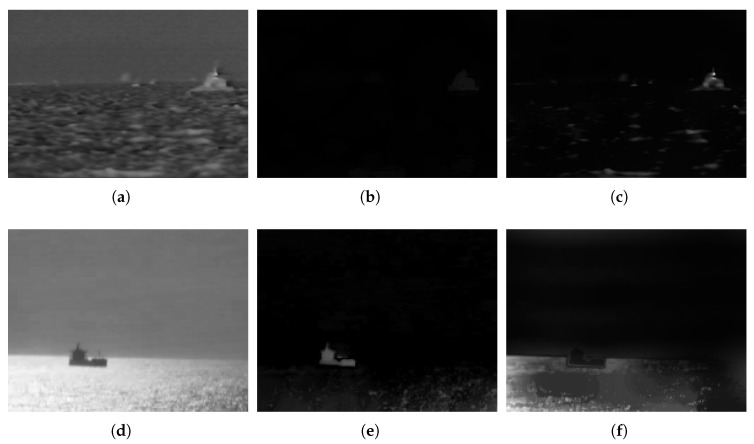
Results of morphological reconstruction in two scenes. (**a**) Original grayscale image of scene 1. (**b**) Dilation-based reconstruction result of scene 1. (**c**) Erosion-based reconstruction result of scene 1. (**d**) Original grayscale image of scene 2. (**e**) Dilation-based reconstruction result of scene 2. (**f**) Erosion-based reconstruction result of scene 2.

The reconstruction results are shown in Figure 2. There are two different scenes and each of them has a ship target with different features. Figure 2a is the original grayscale image including a bright target. The ER of Figure 2a, shown as Figure 2c, accurately preserves the overall contour of the ship and eliminates most of the sea clutter, while nearly nothing remains in the DR result, as can be seen in Figure 2b. Similarly, Figure 2d shows another scene that includes a dark ship target. The DR operation, shown in Figure 2e enhances the dark target and reduces the brightness of the surrounding pixels, while the ER operation poorly enhances the dark ship target, as can be seen in Figure 2f. Thus, we can conclude that the ER(DR) operation removes the dark(bright) background and helps to enhance the bright(dark) targets. In existing research, some studies [28,29] are based on the assumption that bright and dark targets are equally likely to appear in an image. This assumption may be closer to the actual situation, but it also increases the possibility of complex backgrounds being misjudged as ship targets. Thus, in this paper, we take the ER operation as the basis for further research on bright ship target segmentation.

### 2.2. Multiscale Saliency Map

Although current methods have achieved great success in the field of ship target detection through morphological reconstruction methods, most of them simply use a structuring element of a specific scale during the reconstruction process but ignore the key issue that a different scale structuring element may lead to a different segmentation result. Thus, it is important, and necessary, to choose an appropriately sized structuring element.

As the ship targets usually have various sizes, it is hard to determine a single structuring element that is effective in all scenarios. Thus, we propose a multiscale morphological reconstruction (MMR) method that uses several structuring elements with different scales at the same time to process a grayscale image. The forms of multiscale dilation-based reconstruction (MDR) and multiscale erosion-based reconstruction (MER) are defined in Equations (6) and (7), respectively.

(6) $$\mathrm{MDR}\left(g,s,e\right)=\wedge_{s\leq i\leq e}\left\{DR_{g}{\left(f\right)}_{b_{i}}\right\}$$

(7) $$\mathrm{MER}\left(g,s,e\right)=\vee_{s\leq i\leq e}\left\{ER_{g}{\left(f\right)}_{b_{i}}\right\}$$

where $\left\{b_{i}\right\}$ is a series of nested structuring elements. The value *s* denotes the minimum scale value of the structuring element and *e* denotes the maximum scale value of the structuring element, 1≤s≤i≤e,*s*, *i*, *e*∈$N^{+}$. In our method, *s* should be small so that small targets can be detected in the result. The value of *s* should be between 1 to 5. Similarly, the value of *e* should be relatively large so that targets with large sizes can be segmented completely. The value of *e* should be between 10 to 20. *f* is the marker image; and *g* is the mask image. The MR operation helps reconstruct the grayscale image while improving the salience of different-sized targets.

By combining MDR and MER, we obtain a multiscale foreground map (MFM) that ensures the ship targets are more notable in the image and reduces other interference, such as sea clutter and environmental noise.

(8) $${\mathrm{MFM}}_{b_{i}}={\mathrm{TH}(\mathrm{MER})}_{b_{i}}+{\left\|\mathrm{MDR}-g\right\|}_{1}$$

where $\mathrm{TH}\left(\cdot \right)$ is the Top-Hat operation to further extract foreground regions and ${\left\|\cdot \right\|}_{1}$ is the $\ell_{1}-norm$ operation to enable the differences between MDR and the original image to be more notable. As analyzed in Section 2.1, the MER operation enhances the intensity of bright targets, so we selected the Top-Hat operation to further highlight the bright targets. By subtracting MDR from the original image, the dark region is further suppressed. Note that the MFM is also related to the structuring element $b_{i}$; we can integrate different scales of $b_{i}$ by assigning them different weights. Thus, the integrated multiscale foreground map (IMFM) is given as follows.

(9) $$\mathrm{IMFM}=\frac{1}{e}\sum_{i=1}^{e}\omega_{i}\cdot {\mathrm{MFM}}_{b_{i}}$$

where $\omega_{i}$ is the weight for the *i*th scale, and is chosen depended on the image size.

To clearly observe an enhanced result, we convert the grayscale image IMFM into a binary image BIMFM through an adaptive threshold,

(10) BIMFM=μ+k×σ

where μ and σ are the mean value and variance of IMFM, respectively. Furthermore, *k* is an empirical constant that controls the proportion of the variance, and the value of *k* can be selected from the interval $\left[1,5\right]$ for most scenarios.

**Figure 3:**
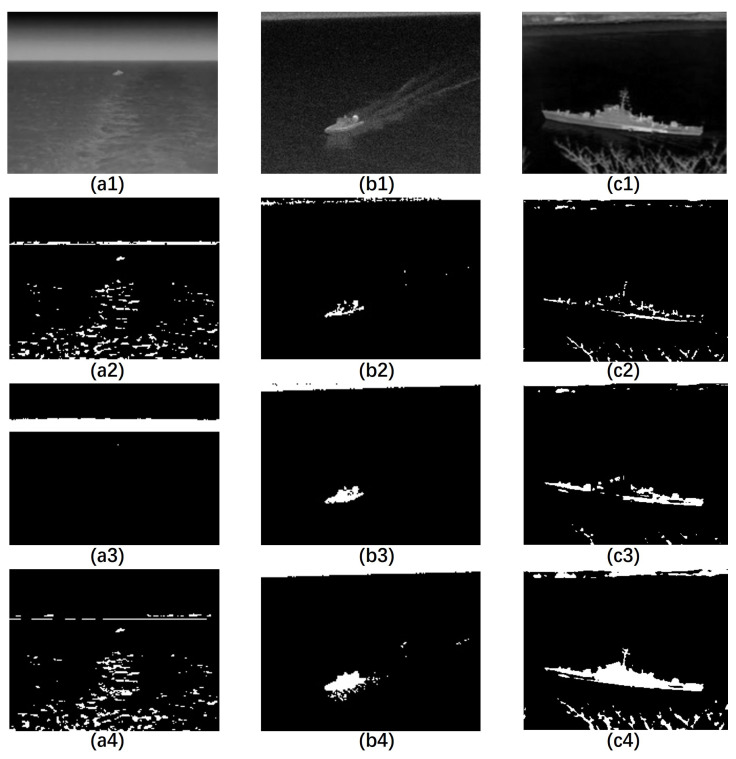
Results of reconstruction with different scales of the structuring element. (**a1**,**b1**,**c1**) are the original grayscale images. (**a2**,**b2**,**c2**) are the binarized ER results with a scale 5 structuring element. (**a3**,**b3**,**c3**) are the binarized ER results with a scale 20 structuring element. (**a4**,**b4**,**c4**) are the BIMFM results.

From Figure 3, it is clear that for small targets, such as those in Figure 3(a1–a4), a small scale structuring element achieved good performance in reconstruction and segmentation. However, when the scale value increased to 20, we failed to find the target and the most notable part remaining in the image was the sky–sea line. Moreover, for larger targets, such as those in Figure 3(b1–b4,c1–c4), a small-scale structuring element could not restore the target completely. By introducing the proposed IMFM method, we achieved good reconstruction and segmentation results for both small and large targets.

### 2.3. Feature-Based Structure Tensor

Although we achieved significant results in segmenting ship targets, there was still some interference remaining in the binary image. Therefore, we needed to take further measures to process the image so that only the ship targets remained in the end. The use of the structure tensor (ST) [30], based on the edge shock filter and variational functionals, is very effective in enhancing corner structures and presents different characteristics in homogeneous regions, edges, and texture regions of an image. In [31], Zhang et al. proposed a multi-directional structure tensor to detect corners in a picture. In [32], Wang et al. constructed a weighted directional structure tensor to overcome the fuzzy appearance problem of the anisotropic diffusion filtering algorithm. In our method, we adopt a structure tensor to extract the contour information of ship targets. Given a grayscale image *g*, the structure tensor ST of *g* can be computed as follows:

(11) $$ST=F_{\sigma }★\left(▽g▽g^{T}\right)=\left[\begin{array}{cc} F_{\sigma }★g_{x}^{2} & F_{\sigma }★g_{x}g_{y}\\ F_{\sigma }★g_{y}g_{x}\; & F_{\sigma }★g_{y}^{2} \end{array}\right]=F_{\sigma }★G$$

where $F_{\sigma }$ is a filter with standard deviation σ, i.e., a Gaussian filter; ★ is the convolution operator; and $▽g={\left(g_{x},g_{y}\right)}^{T}$ represents the image gradient. The structure tensor matrix ST can be seen as the convolutional result of the template filter $F_{\sigma }$ and the gradient matrix *G*. Note that, in the real domain R, $g_{x}=g_{y}$, the structure tensor matrix ST can be rewritten as follows:

(12) $$ST=\left[\begin{array}{cc} s1 & s2\\ s2\; & s3 \end{array}\right]$$

The two eigenvalues of matrix ST, denoted as $\lambda_{1}$ and $\lambda_{2}$, are given by the following equation:

(13) $$\lambda_{1,2}=\frac{1}{2}\left(s_{1}+s_{3}\pm \sqrt{{\left(s_{1}-s_{3}\right)}^{2}+4s_{2}^{2}}\right),\lambda_{1}\geq \lambda_{2}$$

The large eigenvalue $\lambda_{1}$ of the structure tensor, denoted as STLE (structure tensor’s large eigenvalue), indicates the predominate direction and the coherence degree of the gradient trend. The structure tensor’s small eigenvalue (STSE), denoted as $\lambda_{2}$, is usually highlighted in the pixels that represent the most notable corner regions [33]. In the previously obtained IMFM, as the targets were enhanced and were markedly different from the adjacent environment, the intensity of $\lambda_{2}$ of the targets was much larger than for other interference. So, in our method, we take IMFM as the image *g* in Equation (11) for the subsequent processing.

**Figure 4:**
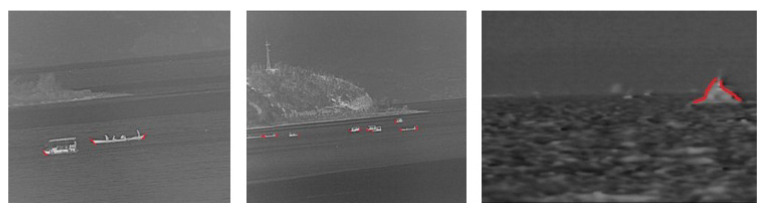
Examples of the common features of ships.

**Figure 5:**
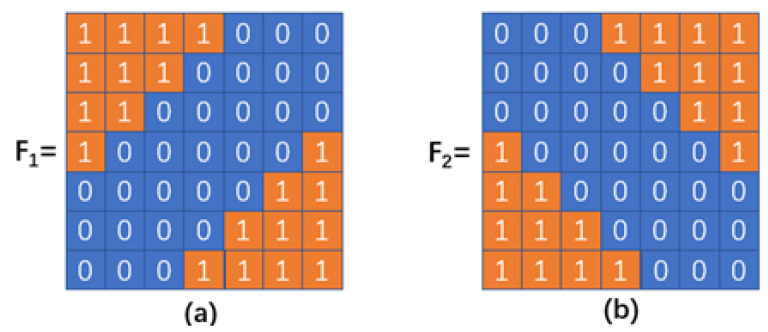
Two designed feature-based filer templates. (**a**) Filter template 1 that highlights the upper left direction edge feature. (**b**) Filter template 2 that highlights the lower right direction edge feature.

As ship targets vary in shape and size, it is hard to determine a common expression to describe all types of ship targets. However, we can form an approximate representation of the characteristics of ship targets. For example, they are flat at the bottom and have an obvious steepness in the vertical direction, which is determined by the nature of the ship, as can be seen in Figure 4. Ship targets in Figure 4 have common ground at the bottom (or top), and a significant change in the gradient. Therefore, we designed two templates, based on these features, and applied them to the filter in Equation (12) to further improve the contrast between the targets and the background. In doing this, we discriminate ship targets from other noise that still remains in the IMFM. The  feature-based filter templates are shown in Figure 5.

As can be seen in Figure 5, the templates are highlighted in the corner region and we set the value of the part where the edges rise to 1 and to 0 for the rest. By utilizing this unique attribute, that other noise does not have, the edges of ship targets are emphasized. By convolving the filter and the ST matrix, i.e., that ${\mathrm{STME}}_{1}=F_{1}★G$ and ${\mathrm{STME}}_{2}=F_{2}★G$, we obtain the feature-based structure tensors, as well as their corresponding small eigenvalues, ${\mathrm{STSE}}_{1}$ and ${\mathrm{STSE}}_{2}$. The feature-based contour map (FCM) is constructed as shown in Equation (14).

(14) $$\mathrm{FCM}=\mathrm{STLE}+{\mathrm{STSE}}_{1}+{\mathrm{STSE}}_{2}$$

where STLE depicts the general outline and ${\mathrm{STSE}}_{1}$ and ${\mathrm{STSE}}_{2}$ enhance the corner intensity to further emphasize the ship targets.

**Figure 6:**
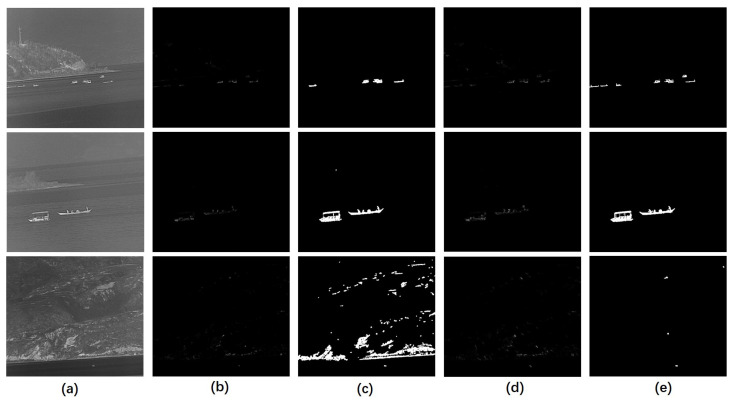
An example of the performance of the FCM. (**a**) The original grayscale images. (**b**) The FCM of the original images. (**c**) Binarization result of (**b**). (**d**) Contour map without introducing the feature-based template. (**e**) Binarization result of (**d**).

Figure 6 shows the performance of the FCM. There are three scenes in Figure 6 and each of them contains a number of ship targets with different scales. By introducing the FCM, the ship targets were more notable in the infrared image than before, while the background, such as the shore and mountains, was suppressed, appearing darker. However, if we did not introduce the feature-based template to convolve with the structure tensor, as shown in Figure 6d,e, situations where the target was missing, excessive noise introduced, and a large number of mountain backgrounds not eliminated, were possible. The latter situations did not show up once we introduced the FCM. We calculated the average FCM of the contours in Figure 6 by dividing the FCM value by the perimeter of the contours. Then, we took 50% of the maximum value as a threshold to further filter the segmentation result. As shown in Figure 6d, all ship targets were retained in the binarized result and nearly no background interference remained.

### 2.4. Two-Branch Compensation Strategy

**Figure 7:**
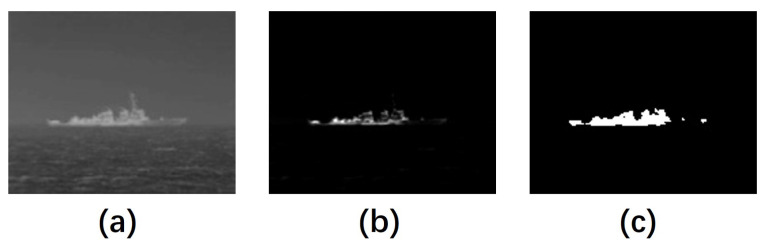
An example of uneven distribution of a ship. (**a**) The grayscale image. (**b**) The IMFM of (**a**). (**c**) The segmentation result.

In some scenarios, usually seen in cases where ship targets are large, the overall gray distribution of the ship targets is uneven. This presents a problem in the segmentation stage, where part of the ship target may not be recognized correctly and the target may be incomplete in the segmentation result, as shown in Figure 7.

**Figure 8:**
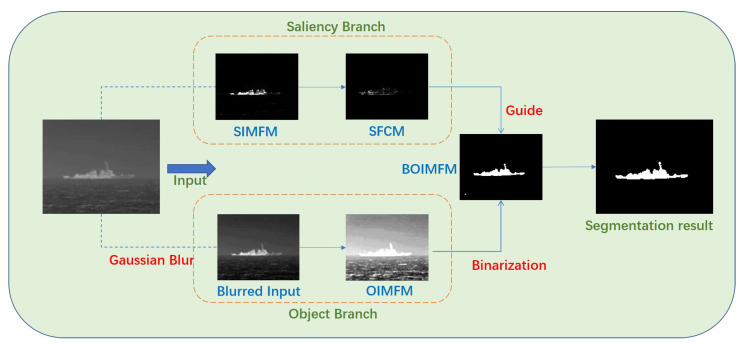
Overview of the two-branch compensation strategy.

To address this problem, we designed a two-branch compensation strategy, based on previous research [28,34]. To be specific, we divided the original grayscale image into two branches: an object branch and a saliency branch. The object branch highlights the general outline of the target, and the saliency branch reflects the most prominent part of the original image. Figure 8 shows how this strategy works. The saliency branch is generated by the original grayscale image. Following a previous process, we first applied multiscale morphological reconstruction to the original grayscale image and obtained the saliency-branch integrated multiscale foreground map (SIMFM). Then, by calculating the FCM of the SIMFM, we obtained the saliency-branch feature-based contour map (SFCM) as the guiding image. We adopted Gaussian blur, [35], to process the input grayscale image in the first stage. Gaussian blur is a widely used effect in the image preprocessing stage, typically to reduce image noise and reduce detail. By applying Gaussian blur, we enhanced image structures at different scales. We obtained blurred input, which had less noise but also less details, compared to the original image. As can be seen, the uneven distribution was eliminated to some extent. Then, we obtained the binarization result of the object branch IMFM (OIMFM), denoted as BOIMFM. Under the guidance of the SFCM, the remaing noise in the BOIMFM was removed and the target was segmented completely in the segmentation result, despite the uneven grayscale distribution in the original image. The implementation of the proposed multiscale morphological reconstruction-based saliency mapping, combined with two-branch compensation strategy (MMRSM-TBC), method is shown in Algorithm 6.
**Algorithm 1:** MRMSP-TBC**Input:** The original infrared image, *I*;**Output:** The detection binarized result of *I*;1:Compute the multiscale morphological reconstruction results, according to Equations (6) and (7);2:Calculate the multiscale foreground map (MFM) of scale $b_{i}$, according to (8);3:Integrate the multiscale foreground map (MFM) by setting different weights and obtain the integrated multiscale foreground map (IMFM), according to Equations (8) and (9);4:Derive the structure tensor (ST) of IMFM, according to Equation (11), and calculate the large eigenvalue (STLE), according to Equation (13);5:Initialize two feature-based templates, $F_{1}$ and $F_{2}$, according to Figure 5;6:Convolve the two templates with ST and obtain two new ST matrices, $S_{1}$ and $S_{2}$;7:Derive the small eigenvalue of $S_{1}$ and $S_{2}$, denoted as $STSE_{1}$ and $STSE_{2}$, respectively, according to Equation (13);8:Calculate feature-based contour map (FCM) by combining STLE and STSE, according to Equation (14);9:Obtain the saliency-branch feature-based FCM (SFCM) and the maximum value of SFCM, denoted as M;10:Process the original infrared image with Gaussian blur as the blurred input of the object branch;11:Obtain the IMFM of the blurred input (OIMFM);12:Obtain the Binarized OIMFM (BOIMFM), according to Equation (10), and count the number of detected targets, denoted as N in BOIMFM;13:**for** detected target number n=1:N **do
**14: Compute the average value of SFCM of the n-th detected target;15: Eliminate the n-th detected target if the avaerage value of SFCM is smaller than 0.5*M;16:**end for**17:Obtain the BOIMFM under the guidance of SFCM as the final detected result, R;18:**return** *R*;

## 3. Experiments

In this section, we assessed our proposed method’s effectiveness through experiments. We collected nine sequences of infrared ship images, each sequence having its own characteristics. The nine sequences comprised a total of 793 infrared images. The experiments were performed on a platform with an Intel i7-11700KF, 3.6 GHz CPU, and 16 GB memory.

### 3.1. Test Dataset

We tested our method on nine sequences, with each sequence containing a different number of targets with different sizes. We defined the target as small, medium and large, using the following definition: when the target size is smaller than 0.03*w*h, where w is the width of the original image and h is the height of the original image, the target is of a small size. Similarly, when the size of the target is between 0.03*w*h and 0.1*w*h, we define the size of the target as medium. A target which has a size larger than 0.1*w*h, is defined as a large target. For example, Seq2 had a total of eight ship targets with small and medium sizes, while there was only one target in the image of Seq6 and its size was very small. The details of the sequences are listed in Table 1.

### 3.2. Discussion of Key Parameters

As described in the introduction above, there are four key parameters in our algorithm: the minimum scale value of the structural element B, denoted as *s* in Equations (6) and (7); the maximum scale value of the structural element B, denoted as *e* in Equations (6) and (7); the weight $\omega_{i}$ in Equation (9); the constant *k* in Equation (10) to binarize the saliency map. In order to detect small targets and, at the same time, find large targets with prominent contours, we set *s* as 3 and *e* as 15. As the target size was unknown during detection, we simply set $\omega_{3}$ and $\omega_{15}$ as 2 and the rest as 1 to cover all target sizes. The parameter *k* was chosen empirically to be 1.5, which demonstrated good performance in binarization.

### 3.3. Qualitative Comparison

We compared our proposed method with a few classical algorithms that are commonly used for target detection and several advanced algorithms that have been used for ship target detection in recent years, covering the main concepts of morphological filtering, local-characteristics-based detection, and multiscale transformation-based detection. To be specific, we chose the following to test the infrared images: Top-Hat filter [36], multiscale tri-layer LCM(TLLCM) [37], partial sum of the tensor nuclear norm (PSTNN) [36], morphological-reconstruction-based multifeature analysis(MRMFA) [16], two-channel image separation combined with saliency mapping of local grayscale dynamic range (TCS-SMoLGDR) [28], improved FCM algorithm based on morphological reconstruction and membership filtering (FRFCM) [24], KL divergence-based FCM (KLDFCM) [26].

**Figure 9:**
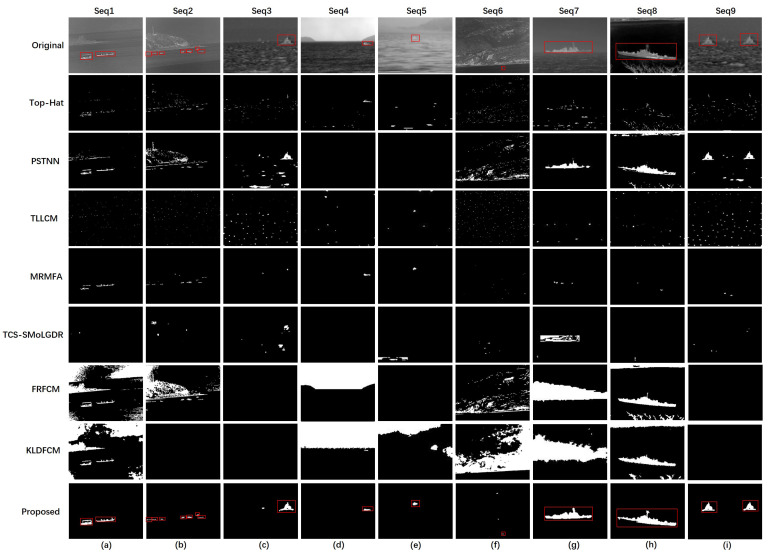
Detection results of different methods. (**a**–**i**) The original image of (Seq1–Seq9) and the corresponding detection results of the methods used for comparison.

Figure 9 shows the detection results of different methods on the test dataset. In the original image, each target is highlighted by a red box and we also highlight the detected targets in the results on our proposed method. It is clear that our method achieves a great performance in detecting infrared ship targets. For detailed analysis, the Top-Hat filter, which is a classical method for suppressing background and enhancing targets, could roughly extract the targets. However, when the contrast between the target and the background was low, such as in Seq5, the target was barely detected using Top-Hat. The TLLCM method aims to estimate the background and further extract the target. However, TLLCM could only correctly find the target in Seq5, in which the target was small. This was because the target sizes varied and the method could not estimate the background well. PSTNN could restore the contour of the targets completely, but, at the same time, it also introduced much background noise, such as the shore, mountains and sea clutter. When detecting small targets, PSTNN did not work well. MRMFA exhibited good performance when detecting targets in a flat background. However, it had one drawback, which was that, when the background was complex, it might misjudge the background as a target and might even fail to detect a target, such as in the Seq6 scene. Furthermore, when the grayscale distribution of the target was uneven, it could not recover the target completely, as shown in Figure 9h. TCS-SMoLGDR hardly detected a target in our test dataset. This was because TCS-SMoLGDR uses sliding windows to extract the target, but when the contrast between the target and background is low, it cannot distinguish targets from the background. The FRFCM performed well in segmenting large interconnected areas, such as those in Seq1 and Seq2, but the issue was that, although the ship targets were segmented completely, the background was also introduced, which made it more difficult to detect the ship targets. This was because the FRFCM is a Fuzzy C Means-based algorithm and does not take into account the objective distribution of objects. Another MR-based method, KLDFCM, has the same issue, in that it is only effective when segmenting large targets, such as in Seq8. The KLDFCM also has a problem in that it costs a lot of time for the iteration to reach the stop condition. By comparison, it can be seen that our proposed method performed well in different scenarios, including the shore, mountains and strong sea waves. For example, in Seq3, there was severe sea clutter and the detection result of PSTNN contained a lot of segmented sea clutter. What is more, in Seq1, our method segmented the two ship targets in the scene and no background was introduced, compared to PSTNN, FRFCM and KLDFCM. When the sea–sky line was present in an image, such as in Seq4, our method did not need to remove the sea–sky line before detection and the target was still located. When the target was significantly small, compared to the background, such as in Seq6, our method successfully distinguished the small target and only misjudged two other small points to be targets, while other methods, such as PSTNN, failed to find the target. In addition, our proposed method was also adaptable to changes in the size of the target. When detecting small targets, our method correctly found them, and when detecting large targets, our method extracted their contours and shapes effectively. In Seq8, although the segmentation result was not as complete as those for FRFCM and KLDFCM, only the target remained in the result, while the other methods introduced extra sky-line and water plants in the results. Furthermore, we did not have to limit the number of targets, since all of them could be detected. In Seq2 there were 8 targets in total and our methods detected all of them, unlike the other methods, which either detected fewer targets or introduced additional background interference.

### 3.4. Quantitative Comparison

In order to quantitatively evaluate the detection performance of the selected methods, we chose four metrics to conduct a quantitative comparison: the misclassification error (ME) [38], the true positive rate (TPR) [28], the false positive rate (FPR) [28] and the intersection over union (IoU) [39]. The meanings and expressions of the metrics are listed as follows:

(15) $$\mathrm{ME}=1-\frac{\left|B_{GT}⋂B_{DR}\right|+\left|F_{GT}⋂F_{DR}\right|}{\left|B_{GT}\right|+\left|F_{GT}\right|}$$

where $B_{GT}$ and $B_{DR}$ are the background pixels of the ground truth image and the detected result, respectively. Pixels $F_{GT}$ and $F_{DR}$ are the foreground pixels of the ground truth image and the detected result, respectively. The ME denotes the rate with which the foreground and the background are incorrectly classified. A small ME value represents a lower number of errors in the result and, thus, better detection performance of the algorithm.

(16) $$\mathrm{TPR}=\frac{TP_{DR}}{P_{GT}}$$

(17) $$\mathrm{FPR}=\frac{FP_{DR}}{P_{DR}}$$

where $TP_{DR}$ and $FP_{DR}$ are the number of true positive pixels and false positive pixels of the detected result, respectively. Pixels $P_{GT}$ and $P_{DR}$ are the number of pixels of the ground truth and detected result, respectively. It is clear that TPR denotes the accuracy of segmentation and FPR denotes the proportion of incorrect pixels in the segmentation result. The larger the TPR and the smaller the FPR, the better the detection performance of the method.

(18) $$\mathrm{IoU}=\frac{\left|F_{GT}⋂F_{DR}\right|}{\left|F_{GT}⋃F_{DR}\right|}$$

where $F_{GT}$ is the ground truth image and $F_{DR}$ is the detected result. IoU denotes the the size of the overlapping part of the two images and a large IoU indicates that the method achieves a better segmentation result.

The result of the metrics of the selected methods are listed in Table 2, where a bold number indicates that the method achieves the best performance among all methods used for comparison. Table 2 shows that the proposed method obtained smaller average values for ME and FPR, and, at the same time, obtained larger values for TPR and IOU. The experimental results indicate that the proposed method detected ship targets with different scales and also worked stably in different complex backgrounds.

## 4. Conclusions and Future Work

In this paper, we propose a multiscale morphological reconstruction-baseddetection method to detect infrared ship targets of various scales in complex environments. Our paper proposes a multiscale saliency map to improve the contrast in the infrared images to better highlight the ship targets, while suppressing complex backgrounds. By utilizing two feature-based templates to convolve with the structure tensor, ship target contours are extracted from the image to judge if the detected result is a target or not. Finally, a strategy that divides the image processing procedure into two branches is proposed to prevent the problem of incorrect segmentation caused by uneven grayscale distribution. The experimental results showed that, compared to other methods, our method demonstrates strong performance with regard to visual effects and objective evaluation metrics.

Although the proposed method has achieved significant detection results, as was mentioned before, it is based on the assumption that the grayscale value of the ship target is higher than that of its surrounding background. However, in reality, this is not always true. Due to interference caused by the infrared imaging distance and sunlight reflections, ship targets may appear dark in an image [29]. Therefore, studying how to simultaneously detect bright and dark ship targets, without introducing additional interference, is an important research direction in our future work. In addition, simply using single-frame target detection does not easily adapt to all situations, and it is difficult to avoid false alarms caused by clutter similar to ship gray distribution. We plan to introduce multi-frame detection [40] into our method to assist in identifying false targets in a single image, while also introducing depth feature information [41,42] of an image to enhance the distinguishability of ship targets.

## Figures and Tables

**Table 1:** Details of the test image sequences.

Sequences	Seq1	Seq2	Seq3	Seq4	Seq5	Seq6	Seq7	Seq8	Seq9
Target size	Medium, Large	Small, Medium	Large	Medium	Small	Small	Large	Large	Large
Background	Coast	Mountain	Sea wave	Sea wave	Sea wave	Mountain	Sea wave	Tree	Sea wave
Target Number	2	8	1	1	1	1	1	1	2

**Table 2:** Detection performance of different methods on the test dataset.

Metrics	Methods	Seq1	Seq2	Seq3	Seq4	Seq5	Seq6	Seq7	Seq8	Seq9	Average
ME	Top-Hat	0.5270	0.3715	0.8774	0.2239	1.0000	0.5283	0.7109	0.8369	0.8779	0.6615
	TLLCM	0.9059	0.9180	0.9954	1.0000	0.2632	0.5283	0.8707	0.9190	0.9591	0.8177
	PSTNN	0.2848	0.2628	0.1816	1.0000	1.0000	0.8302	0.1406	0.1870	0.1494	0.4485
	TCS-SMoLGDR	1.0000	0.9662	0.7069	1.0000	1.0000	1.0000	0.4184	1.0000	0.9465	0.8931
	MRMFA	0.4993	0.3715	0.9290	0.1343	0.2105	0.2830	0.8821	0.9749	1.0000	0.5872
	FRFCM	0.1588	**0.1132**	1.0000	1.0000	1.0000	0.3396	0.0011	**0.0743**	1.0000	0.5208
	KLDFCM	0.2274	1.0000	1.0000	0.4328	1.0000	0.7358	**0.0000**	0.0942	1.0000	0.6100
	Proposed	**0.0776**	0.1457	**0.1097**	**0.0746**	**0.0526**	**0.0943**	0.0261	0.1321	**0.0881**	**0.0890**
TPR	Top-Hat	0.4730	0.6285	0.1226	0.7761	0.0000	0.4717	0.2891	0.1631	0.1221	0.3385
	TLLCM	0.0941	0.0820	0.0046	0.0000	0.7368	0.4717	0.1293	0.081	0.0409	0.1823
	PSTNN	0.7152	0.7372	0.8184	0.0000	0.0000	0.1698	0.8594	0.8130	0.8506	0.5515
	TCS-SMoLGDR	0.0000	0.0338	0.2931	0.0000	0.0000	0.0000	0.5816	0.0000	0.0535	0.1069
	MRMFA	0.5007	0.6285	0.0710	0.8657	0.7895	0.7170	0.1179	0.0251	0.0000	0.4128
	FRFCM	0.8412	**0.8868**	0.0000	0.0000	0.0000	0.6604	**0.9989**	0.9257	0.0000	0.4792
	KLDFCM	0.7726	0.0000	0.0000	0.5672	0.0000	0.2642	0.5509	**1.0000**	0.9058	0.0000
	Proposed	**0.9224**	0.8543	**0.8903**	**0.9254**	**0.9474**	**0.9057**	0.9739	0.8679	**0.9119**	**0.9110**
FPR	Top-Hat	0.3334	0.8420	0.9380	0.8506	1.0000	0.9984	0.7319	0.7048	0.8961	0.8106
	TLLCM	0.9456	0.9828	0.9940	1.0000	0.9397	0.9982	0.6715	0.6701	0.9767	0.9087
	PSTNN	0.5655	0.9254	0.7741	1.0000	1.0000	0.9996	0.1444	0.5008	0.4222	0.7036
	TCS-SMoLGDR	1.0000	0.9613	0.5825	1.0000	1.0000	1.0000	0.2338	1.0000	0.6027	0.8200
	MRMFA	**0.0162**	0.3682	0.3851	0.4328	0.6500	0.9426	0.2332	0.0393	1.0000	0.4519
	FRFCM	0.9447	0.9641	1.0000	1.0000	1.0000	0.9991	0.5509	0.5903	1.0000	0.8943
	KLDFCM	0.9479	1.0000	1.0000	0.9952	1.0000	0.9999	0.6547	0.5430	1.0000	0.9045
	Proposed	0.0360	**0.2057**	**0.1949**	**0.3841**	**0.5915**	**0.8399**	**0.1250**	**0.0142**	**0.0891**	**0.2756**
IoU	Top-Hat	0.3041	0.1032	0.0362	0.0934	0.0000	0.0014	0.1325	0.1018	0.0497	0.0914
	TLLCM	0.0296	0.0103	0.0026	0.0000	0.0378	0.0017	0.0862	0.0638	0.0122	0.0271
	PSTNN	0.2944	0.0508	0.1843	0.0000	0.0000	0.0002	0.5557	0.3983	0.4314	0.2128
	TCS-SMoLGDR	0.0000	0.0179	0.1485	0.0000	0.0000	0.0000	0.2895	0.0000	0.0491	0.0561
	MRMFA	0.4381	0.3426	0.0659	0.4056	0.2344	0.0486	0.1071	0.0248	0.0000	0.1852
	FRFCM	0.0420	0.0239	0.0000	0.0000	0.0000	0.0008	0.1493	0.3446	0.0000	0.0623
	KLDFCM	0.0386	0.0000	0.0000	0.0027	0.0000	0.0001	0.1138	0.3543	0.0000	0.0566
	Proposed	**0.6993**	**0.5038**	**0.6531**	**0.4336**	**0.2500**	**0.1429**	**0.5808**	**0.7659**	**0.7011**	**0.5256**

## Data Availability

The data that support the findings of this study are available from the corresponding author upon reasonable request.
